# Automated prediction of emphysema visual score using homology-based quantification of low-attenuation lung region

**DOI:** 10.1371/journal.pone.0178217

**Published:** 2017-05-25

**Authors:** Mizuho Nishio, Kazuaki Nakane, Takeshi Kubo, Masahiro Yakami, Yutaka Emoto, Mari Nishio, Kaori Togashi

**Affiliations:** 1 Department of Diagnostic Imaging and Nuclear Medicine, Kyoto University Graduate School of Medicine, Kyoto, Kyoto, Japan; 2 Preemptive Medicine and Lifestyle Disease Research Center, Kyoto University Hospital, Kyoto, Kyoto, Japan; 3 Department of Molecular Pathology, Osaka University Graduate School of Medicine and Health Science, Suita, Osaka, Japan; 4 Department of Medical Science, Kyoto College of Medical Science, Nantan, Kyoto, Japan; 5 Department of Pathology, Kobe University Graduate School of Medicine, Kobe, Hyogo, Japan; Harbin Institute of Technology Shenzhen Graduate School, CHINA

## Abstract

**Objective:**

The purpose of this study was to investigate the relationship between visual score of emphysema and homology-based emphysema quantification (HEQ) and evaluate whether visual score was accurately predicted by machine learning and HEQ.

**Materials and methods:**

A total of 115 anonymized computed tomography images from 39 patients were obtained from a public database. Emphysema quantification of these images was performed by measuring the percentage of low-attenuation lung area (LAA%). The following values related to HEQ were obtained: *nb*_0_ and *nb*_1_. LAA% and HEQ were calculated at various threshold levels ranging from −1000 HU to −700 HU. Spearman’s correlation coefficients between emphysema quantification and visual score were calculated at the various threshold levels. Visual score was predicted by machine learning and emphysema quantification (LAA% or HEQ). Random Forest was used as a machine learning algorithm, and accuracy of prediction was evaluated by leave-one-patient-out cross validation. The difference in the accuracy was assessed using McNemar’s test.

**Results:**

The correlation coefficients between emphysema quantification and visual score were as follows: LAA% (−950 HU), 0.567; LAA% (−910 HU), 0.654; LAA% (−875 HU), 0.704; *nb*_0_ (−950 HU), 0.552; *nb*_0_ (−910 HU), 0.629; *nb*_0_ (−875 HU), 0.473; *nb*_1_ (−950 HU), 0.149; *nb*_1_ (−910 HU), 0.519; and *nb*_1_ (−875 HU), 0.716. The accuracy of prediction was as follows: LAA%, 55.7% and HEQ, 66.1%. The difference in accuracy was statistically significant (*p* = 0.0290).

**Conclusion:**

LAA% and HEQ at −875 HU showed a stronger correlation with visual score than those at −910 or −950 HU. HEQ was more useful than LAA% for predicting visual score.

## Introduction

Chronic obstructive pulmonary disease (COPD) is a leading cause of morbidity and mortality worldwide [1]. COPD causes considerable economic and social burden, which continue to increase. The Global Initiative for Chronic Obstructive Lung Disease guideline defines COPD as a preventable and treatable disease, which is characterized by persistent airflow limitation [2]. The airflow limitation of COPD is usually progressive and associated with an enhanced chronic inflammatory response in the airways and the lung to noxious particles or gases. The airflow limitation is caused by a mixture of small airway disease and emphysema [2], which are often regarded as discrete phenotypes [3].

The percentage of low-attenuation lung area (LAA%) and visual scoring based on computed tomography (CT) images is frequently employed for evaluation of emphysema [3–13]. Although both these parameters are useful for evaluating the severity of emphysema, LAA% has been more frequently used for research purposes owing to the wide availability of software for calculating LAA% and the superior reproducibility of LAA%. However, visual score incorporates information that is not captured by LAA%, such as the spatial distribution of low-attenuation lung regions and findings other than emphysema [8, 9]. For example, visual score was shown to be associated with lung cancer risk in patients with emphysema, although the quantitative measures of emphysema (including LAA%) did not show such an association [10–12]. This implies that visual score may capture more clinically relevant information than LAA%.

In recent years, image processing using homology method is increasingly being used [13–18]. For example, Nishio *et al* used homology method for evaluating the spatial distribution of low-attenuation lung regions in patients with and without COPD [13], and they showed that homology-based emphysema quantification (HEQ) was useful for the assessment of emphysema severity. Because the previous study [9] showed that visual score was affected not only by LAA% but also by the spatial distribution of low-attenuation lung regions, it is conceivable that HEQ could be a more accurate predictor of visual score than LAA%.

The purpose of the current study was to investigate the relationship between visual score and emphysema quantification (LAA% and HEQ) and evaluate whether visual score was accurately predicted by supervised machine learning and emphysema quantification. Previously, a LAA% threshold was optimized by assessing the relationship between LAA% and severity of COPD. To our knowledge, there was no study to investigate the effect of the LAA% threshold on the relationship between LAA% and visual score. For this purpose, LAA% and HEQ were calculated at various threshold levels in the present study. In addition, the combination of emphysema quantification at various threshold levels was used for predicting visual score with supervised machine learning. This method was inspired by persistent homology. Persistent homology is a method for computing topological features at different spatial resolution [19, 20]. Unlike persistent homology, feature vector of the current study was simply constructed using the concatenation of Betti numbers obtained from binarized CT images at the various threshold levels. The method of the current study is similar to those used in bioinformatics, such as Pse-in-One, Pse-Analysis, repDNA, and iDHS-EL [21–24]. These studies and the current study focused on how to create the feature vector which can be easily and effectively combined with machine learning algorithm.

## Materials and methods

The current study used anonymized data from a public database. Therefore, approval of institutional review board or informed consent obtained from patients was not necessary in our country.

### Database of CT images

**Fig 1:**
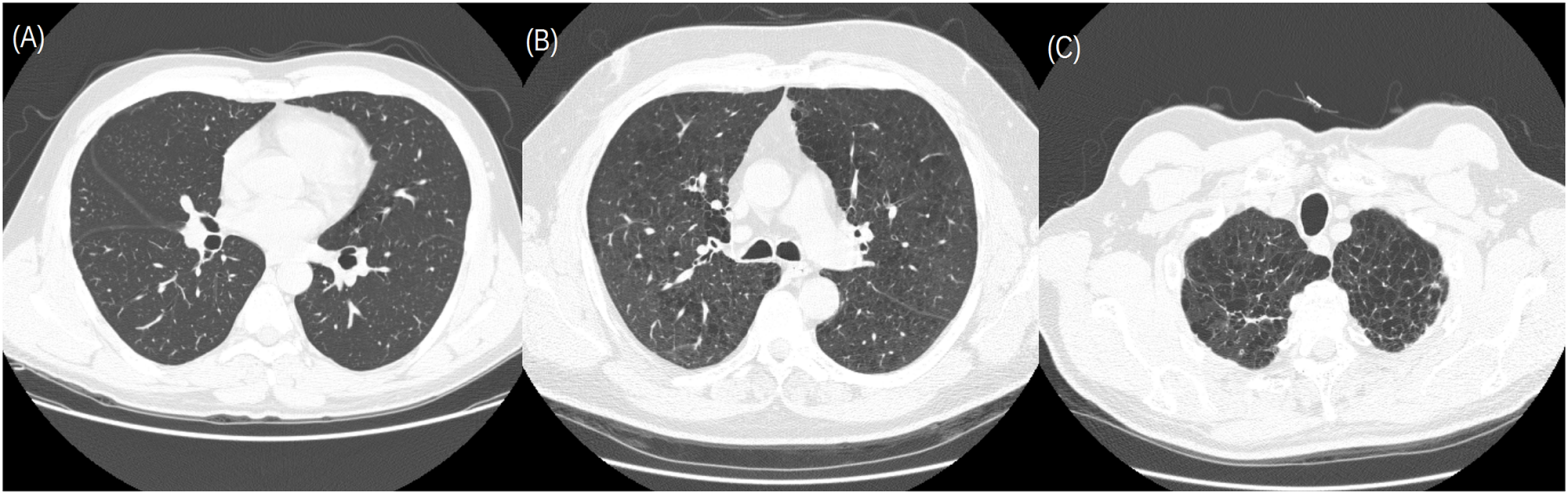
Representative CT images in the database. (A) visual score = 0 (no emphysema); (B) visual score = 3 (moderate); (C) visual score = 5 (very severe). The CT images were displayed with a lung window setting of 1600 HU window width and −550 HU window level.

**Fig 2:**
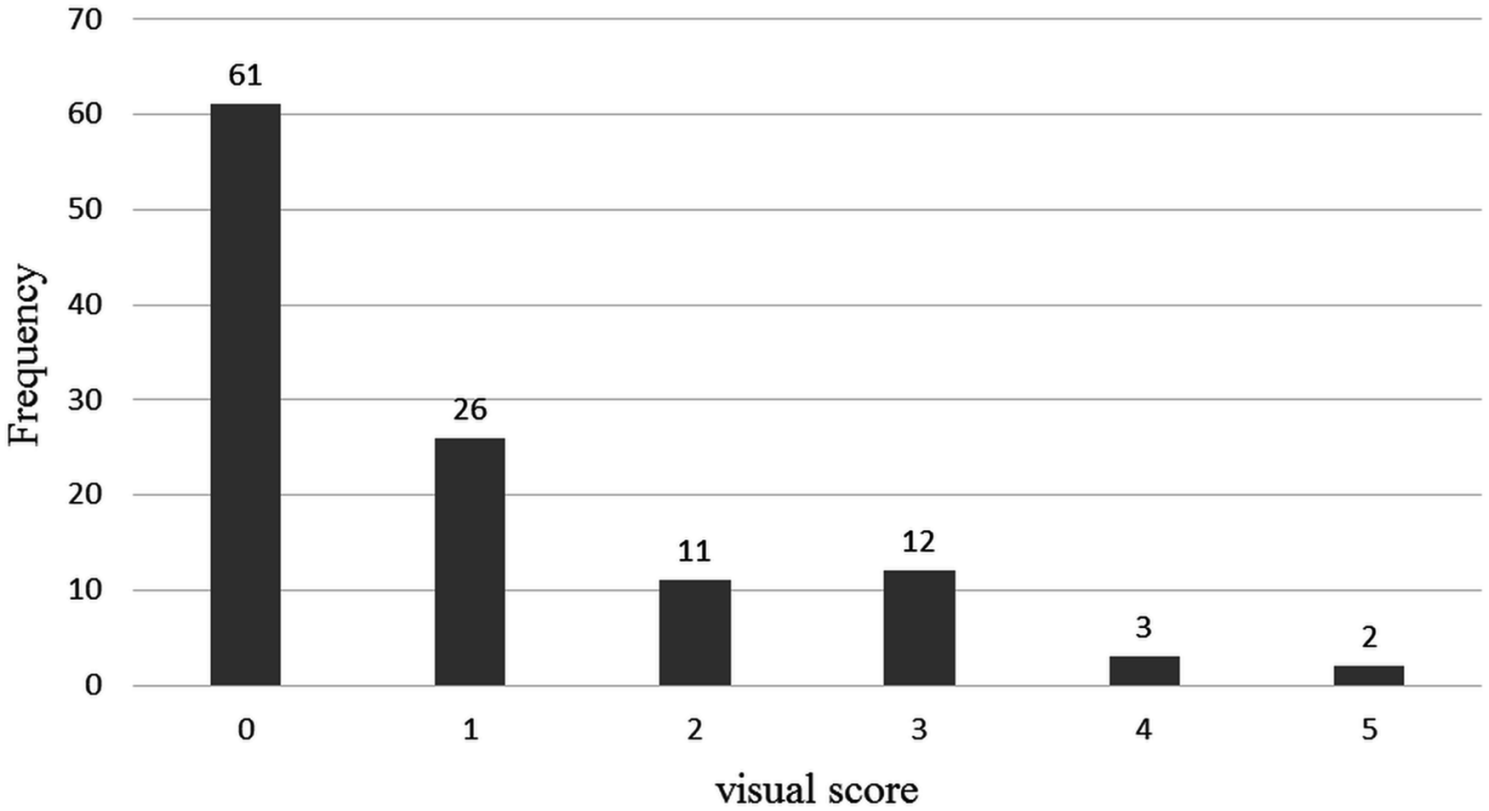
Summary of visual score in the 115 CT slices. Note: Visual score was based on the following criteria: 0, no emphysema; 1, minimal; 2, mild; 3, moderate; 4, severe; and 5, very severe emphysema.

The details of the CT database are available elsewhere [25, 26]. CT images of 39 subjects (9 never smokers, 10 smokers without COPD, and 20 smokers with COPD) were obtained from the database. The CT examinations were performed using four-detector rows CT scanner (LightSpeed QX/i; General Electric Medical Systems, Milwaukee, WI, USA). The following parameters were used: in-plane resolution, 0.78 × 0.78 mm; slice thickness, 1.25 mm; tube voltage, 140 kV; and tube current-time product, 200 mAs. The CT images were reconstructed using a high-spatial-resolution algorithm. The database provided 115 high-resolution CT slices. The severity of emphysema for each of the 115 slices was assessed as visual score by an experienced chest radiologist and a CT experienced pulmonologist. The score criteria were as follows: 0, no emphysema; 1, minimal; 2, mild; 3, moderate; 4, severe; and 5, very severe emphysema. A consensus was reached in case of any disagreement. Representative CT images of the database are shown in Fig 1. Summary of visual score in the 115 CT slices is shown in Fig 2.

### Emphysema quantification

**Fig 3:**
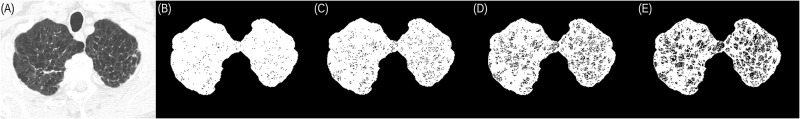
Representative CT and binarized images at multiple threshold levels. (A) CT image; (B)–(E) binarized images at threshold levels of −975, −950, −925, and −900 HU. Note: Fig 3(A) is identical to Fig 1(C). The CT images were displayed with a lung window setting of 1600 HU window width and −550 HU window level.

The methodology for calculation of LAA% and HEQ is described in the previously published papers [4, 13]. First, the lungs were automatically segmented from the CT images based on region-growing method and a threshold of −500 HU. After lung segmentation, LAA% was calculated as follows: $\text{LAA\%}=\;\frac{\text{Total number of low}-\text{attenuation lung pixels }}{\text{Total number of lung pixels}},$ where low-attenuation lung pixels were defined as lung pixels with CT values lower than the predefined threshold [4]. When calculating LAA%, the CT images were binarized using the predefined threshold and results of lung segmentation. In the binarized CT images, 1 indicated a normal lung pixel and 0 indicated a non-lung pixel or low-attenuation lung pixel. Representative images of the binarized CT images are shown in Fig 3. The binarized images were used for HEQ.

Next, HEQ was performed. Betti numbers are important indices in homology and were used as HEQ in a previous study [13]. Betti numbers comprise *b*_0_ and *b*_1_ in case of two-dimensional images. In the current study, *b*_0_ corresponds to the number of low-attenuation lung regions, and *b*_1_ corresponds to the number of normal lung regions surrounded by the low-attenuation lung regions. Intuitively, *b*_0_ and *b*_1_ are related to “holes” formed because of emphysema. Betti numbers could be calculated from the binarized CT images prepared when calculating LAA%. The detailed process of calculating *b*_0_ and *b*_1_ has been described elsewhere [13]. The examples of calculating *b*_0_ and *b*_1_ are available in S1 Fig (Supporting information). Because *b*_0_ and *b*_1_ were affected by size of lung area, *b*_0_ and *b*_1_ were normalized by the total number of lung pixels [13]. These normalized values were referred to as *nb*_0_ and *nb*_1_, and were used as the results of HEQ.

**Fig 4:**
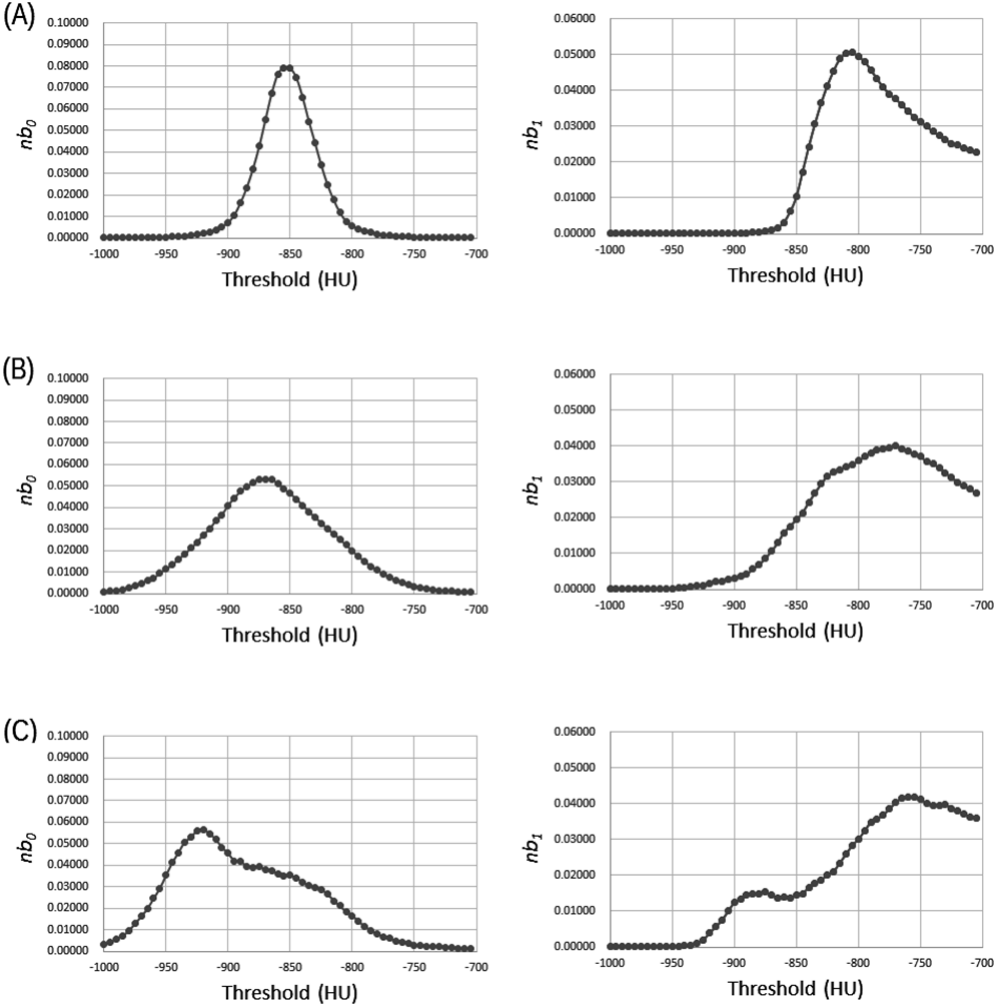
Representative results of HEQ at the 60 threshold levels ranging from −1000 HU to −700 HU. Note: Results of Fig 4(A)–4(C) were obtained from CT images of Fig 1(A)–1(C), respectively. Abbreviation: HEQ, homology-based emphysema quantification; *nb*_0_, the zero-dimensional Betti number normalized by the total number of lung pixel; *nb*_1_, the one-dimensional Betti number normalized by the total number of lung pixel.

LAA% and HEQ were calculated in each of the 115 slices at various threshold levels ranging from −1000 HU to −700 HU. The threshold level was increased in increments of 5 HU. Therefore, LAA% and HEQ was calculated at 60 different threshold levels. Fig 4 shows representative results of HEQ at the 60 different threshold levels, which were obtained from the CT images shown in Fig 1.

### Prediction of visual score using machine learning

Visual score was predicted using supervised machine learning and the results of emphysema quantification (LAA% or HEQ). Random Forest algorithm was adopted for supervised machine learning [27]. As hyperparameters of Random Forest, the following values were used: number of trees in the forest, 10, 100, or 1000; and number of features to consider when searching best split, (length of feature vector) × 0.1, 0.3, 0.5, 0.7, or 0.9. The values of LAA% at the threshold levels ranging from −1000 HU to −700 HU were used as the feature vector of Random Forest, and the classifier for predicting visual score was built. In this classifier (C_LAA%_), the length of feature vector was 60. The other type of classifier was built using Random Forest and the values of *nb*_0_ and *nb*_1_ at the threshold levels ranging from −1000 HU to −700 HU. In the classifier (C_HEQ_), the length of feature vector was 120. For example, for CT images shown in Fig 1(A)–1(C), the feature vector of C_HEQ_ was constructed based on the concatenation of the 1^st^ and 2^nd^ column of Fig 4.

Furthermore, we evaluated the effect of the threshold level on classifiers’ prediction. The lower limit of the threshold was changed from −1000 HU to the following values: −950, −900, −850, −800, and −750 HU. Similarly, the upper limit of the threshold was changed from −700 HU to the following values: −950, −900, −850, −800, and −750 HU. Each combination of the upper and lower limits of the thresholds was evaluated for both C_LAA%_ and C_HEQ_. The length of feature vector was changed based on the lower and upper limits of the threshold. For example, when −1000 and −1000 andere used as the lower and upper limits of the threshold, the length of feature vector of C_LAA%_ was 30.

### Statistical analysis

First, the relationship between emphysema quantification and visual score was evaluated by calculating the Spearman’s correlation coefficient at the various threshold levels. Next, for both C_LAA%_ and C_HEQ_, results of prediction were obtained using leave-one-patient-out cross validation. The best hyperparameters of Random Forest were selected based on the results of the cross validation. To evaluate the performance of C_LAA%_ and C_HEQ_, contingency tables were prepared for the prediction of classifiers and actual visual score based on the results of the cross validation. Then, accuracy of prediction was calculated using the following equation:

$$Accuracy=\frac{TP+TN}{TP+TN+FP+FN},$$

where *TP*, *TN*, *FP*, and *FN* are true positives, true negatives, false positives, and false negatives, respectively. Using the contingency tables of the current study, accuracy was obtained by dividing sum of main diagonal by sum of all elements. The difference in the accuracy between C_LAA%_ and C_HEQ_ was investigated using the McNemar’s test. In addition to accuracy, weighted Kappa was calculated between prediction of classifiers and actual visual score. All statistical analyses were performed using *R*-3.2.2 (available at http://www.r-project.org/). To perform the exact McNemar’s test and calculate the weighted Kappa, exact2x2 package (version-1.4.1) and irr package (version-0.84), respectively, were used. For calculating the weighted Kappa, kappa2 function of irr package was used. “squared” was passed to the kappa2 function as its weight argument.

### Feature selection and others

Because the feature vector obtained in the current study might be redundant, feature selection was performed. The selection was performed based on the importance of the feature calculated by Random Forest. Originally, this method was used in support vector machines, wherein weights of classifier calculated by support vector machines were used as the criteria for the feature selection [28, 29]. The feature selection was performed on the training partitions of leave-one-patient-out cross validation. For each type of the feature vector, the length was reduced by 10%, 30%, and 50% of the original, by using the feature selection. Other types of feature selection and classifier were also evaluated (For the detail, see Supporting information).

## Results

The Spearman’s correlation coefficients for emphysema quantification and visual score at the 60 threshold levels are listed in S1 Table (Supporting information). Table 1 summarizes the results of Spearman’s correlation coefficients. The correlation coefficients were as follows: LAA% at −950 HU, 0.567; LAA% at −910 HU, 0.654; LAA% at −875 HU, 0.704; *nb*_0_ at −950 HU, 0.552; *nb*_0_ at −910 HU, 0.629; *nb*_0_ at −875 HU, 0.473; *nb*_1_ at −950 HU, 0.149; *nb*_1_ at −910 HU, 0.519; and *nb*_1_ at −875 HU, 0.716. For both LAA% and *nb*_1_, the best correlation was obtained at the threshold = −875 HU.

**Table 1:** Spearman’s correlation coefficients for emphysema quantification and visual score.

Threshold (HU)	LAA%	*nb*_*0*_	*nb*_*1*_
−1000	0.410	0.432	−0.170
−975	0.516	0.517	−0.099
−950	0.567	0.552	0.149
−910	0.654	0.629	0.519
−900	0.671	0.616	0.632
−875	0.704	0.473	0.716
−850	0.689	0.095	0.654
−825	0.650	−0.437	0.536
−800	0.600	−0.552	0.333

Note: Spearman’s correlation coefficients at the 60 threshold levels are available in the Supporting information.

Abbreviations: LAA%, percentage of low-attenuation lung area; *nb*_0_, the zero-dimensional Betti number normalized by the total lung pixel; *nb*_1_, the one-dimensional Betti number normalized by the total lung pixel.

Tables 2 and 3 show the accuracy of C_LAA%_ and C_HEQ_ at each combination of the threshold levels, respectively. The best accuracy was as follows: C_LAA%_, 55.7% and C_HEQ_, 66.1%. The best accuracy of C_LAA%_ was obtained when using LAA% at the threshold levels ranging from −1000 HU to −850 HU or from −950 HU to −850 HU. The best accuracy of C_HEQ_ was obtained using *nb*_0_ and *nb*_1_ at the threshold levels ranging from −1000 HU to −700 HU. The difference between the best accuracy of C_LAA%_ and C_HEQ_ was statistically significant (*p* = 0.0290). Tables 4 and 5 show the contingency tables for the most accurate C_LAA%_ and C_HEQ_, respectively. Using the contingency tables provided as Tables 4 and 5, the weighted Kappa was as follows: LAA%, 0.688 and HEQ, 0.697.

**Table 2:** Effect of the threshold level on the predictive accuracy of C_LAA%_ for visual score.

		lower limit of threshold (HU)					
		−1000	−950	−900	−850	−800	−750
upper limit of threshold (HU)	−950	47.0%					
	−900	54.8%	54.8%				
	−850	55.7%	55.7%	51.3%			
	−800	54.8%	53.0%	52.2%	48.7%		
	−750	51.3%	52.2%	48.7%	49.6%	47.8%	
	−700	50.4%	53.9%	49.6%	50.4%	48.7%	47.0%

Note: The best accuracy was 55.7%. Abbreviation: C_LAA%_, classifier using percentage of low-attenuation lung area as feature vector.

**Table 3:** Effect of the threshold level on predictive accuracy of C_HEQ_ for visual score.

		lower limit of threshold (HU)					
		−1000	−950	−900	−850	−800	−750
upper limit of threshold (HU)	−950	52.2%					
	−900	61.7%	59.1%				
	−850	60.9%	63.5%	53.9%			
	−800	63.5%	63.5%	57.4%	54.8%		
	−750	62.6%	65.2%	58.3%	55.7%	54.8%	
	−700	66.1%	63.5%	59.1%	56.5%	53.9%	53.0%

Note: The best accuracy was 66.1%. Abbreviation: C_HEQ_, classifier using homology-based emphysema quantification as feature vector.

**Table 4:** Contingency table for visual score and prediction of C_LAA%._

		prediction					
		0	1	2	3	4	5
visual score	0	48	11	1	1	0	0
	1	15	8	0	3	0	0
	2	4	1	5	1	0	0
	3	1	3	2	3	2	1
	4	0	0	0	2	0	1
	5	0	0	0	2	0	0

Note: Accuracy was 64/115 = 55.7%; Abbreviation: C_LAA%_, classifier using percentage of low-attenuation lung area as feature vector.

**Table 5:** Contingency table for visual score and prediction of C_HEQ_.

		prediction					
		0	1	2	3	4	5
visual score	0	52	6	3	0	0	0
	1	6	17	3	0	0	0
	2	5	2	2	2	0	0
	3	3	1	2	5	0	1
	4	0	0	0	2	0	1
	5	0	0	0	2	0	0

Note: Accuracy was 76/115 = 66.1%. Abbreviation: C_HEQ_, classifier using homology-based emphysema quantification as feature vector.

S2 Table (Supporting information) shows the results of feature selection for C_LAA%_ and C_HEQ_. In both C_LAA%_ and C_HEQ_, there were minimal differences between best accuracy with and without feature selection. This implies either that there was little redundancy in LAA% or HEQ at different thresholds, or that Random Forest could build robust classifiers using LAA% or HEQ even if LAA% or HEQ at the different threshold levels provided redundant information. S3 Table and S1 Doc show the results of other types of feature selection and classifier.

## Discussion

The current study evaluated the relationship between emphysema quantification and visual score. Both LAA% and HEQ showed the strong correlation with visual score; the best correlation coefficients of LAA% and *nb*_1_ were 0.704 and 0.716, respectively. For the correlation between visual score and emphysema quantification, the optimal threshold level for both LAA% and HEQ was −875 HU. When using emphysema quantification and supervised machine learning to predict visual score, HEQ was more useful for predicting visual score than LAA%. The accuracy of C_HEQ_ was statistically better than that of C_LAA%_ (*p* = 0.0290).

The best correlation between LAA% and visual score in our study was observed at the threshold of −875 HU, which was higher than the optimal threshold reported in previous studies. For example, a single LAA% threshold of −950 HU was earlier reported to be an acceptable threshold for emphysema quantification [30]. In previous studies, the LAA% threshold was optimized by assessing the relationship between LAA% and severity of COPD using modalities such as the pulmonary function test. However, we optimized the threshold of LAA% by assessing its relationship with visual score. As a result, the optimal threshold determined in the present study is different from that reported earlier. A previous study [9] suggested that visual score of emphysema was not only determined by LAA% but also by other factors such as lesion size, predominant type, distribution of emphysema, and small-airway disease. These factors may affect the optimal threshold of LAA% determined on the basis of its correlation with visual score.

One clinical application of the current study is to change the threshold of LAA% when lung cancer risk is predicted using CT images. Previous studies have investigated the relationship between emphysema severity (e.g. LAA%) and lung cancer risk using the conventional threshold level (e.g., −950 or −910 HU) [10–12]. These studies showed the significant correlation of visual score of emphysema, but not of LAA%, with the risk of lung cancer. In the present study, the correlation between emphysema quantification and visual score was stronger at the relatively higher threshold level (−875 HU) than the conventional threshold level; therefore, it is speculated that at the relatively high threshold level, LAA% may be significantly associated with the risk of lung cancer. This speculation should be investigated in a larger cohort in future.

Another application of the current study is to utilize the results of C_HEQ_ to predict the risk of lung cancer. Although visual score was significantly associated with the risk of lung cancer, visual score of emphysema can be a severe burden for radiologists or pulmonologists if a lung cancer screening program utilizes CT as a tool for risk stratification. Use of the results of C_HEQ_ in place of visual score may reduce the burden on radiologists or pulmonologists. Because the weighted Kappa between C_HEQ_ and visual score was better than 0.6, C_HEQ_ may potentially be used as a substitute to visual score.

According to Tables 2–5 and the results of the McNemar’s test, the predictive accuracy of C_HEQ_ was statistically better than that of C_LAA%_. In a previous study, HEQ was found useful for evaluating the spatial distribution of low-attenuation lung region [13]. We speculate that because HEQ provides a measure of the spatial distribution of low-attenuation lung region, it may be superior to LAA% for predicting visual score. In our study, use of a wider threshold range improved the predictive accuracy of HEQ (Table 3). This implies that visual score was affected by the spatial distribution of low-attenuation lung region at the relatively high threshold level. This speculation is, at least partially, consistent with the results of a previous study [9].

We used the changes in Betti numbers of the binarized CT images to construct the feature vector for machine learning. Adcock *et al* used intensity filtration and matching metric to utilize support vector machine for classification of liver tumor on CT images [18]. Although their intensity filtration was partly similar to our method, their construction of feature vector was based on the metric of barcode. Qaiser *et al* showed that automated tumor segmentation on histology images could be performed rapidly using topological changes in Betti numbers [31]. Although their method (persistent homology profiles) was compatible with ours, their task was different from ours.

There are several limitations to this study. First, the number of patients was relatively small. In particular, the number of patients with severe or very severe emphysema cases was very small. According to Tables 4 and 5, the predictive accuracy in severe or very severe emphysema cases was worse than that in the other cases. This deterioration in the predictive accuracy may be attributable to the limited number of cases with severe or very severe emphysema. To improve the predictive accuracy and validate the results of the current study, a larger cohort of patients is required for future research. Second, two-dimensional image analyses were performed. Recently, quantification based on thin-slice volumetric CT images has been frequently used. In future, we will extend our method for three-dimensional image analyses. Third, although lung cancer risk was discussed in the current paper, we did not investigate the association between HEQ and the risk. Fourth, although support vector machine with metric or kernel trick specialized in persistent homology was suggested [18, 32], we did not evaluate these methods in the present study. Fifth, the clinical application of HEQ was not investigated in the present study. Because a previous study examined the relationship between HEQ and COPD severity [13], we focused on the relationship between HEQ and visual score of emphysema in the present study.

In conclusion, LAA% and HEQ at −875 HU showed a stronger correlation with visual score as compared to that at the conventional threshold level (−950 or −910 HU). By providing a measure of the spatial distribution of low-attenuation lung region, HEQ was more useful for predicting visual score as compared to LAA%.

## Supporting information

S1 Table Spearman’s correlation coefficients for emphysema quantification and visual score at the 60 threshold levels. (XLS)

S2 Table Results of feature selection in C_LAA%_ and C_HEQ_. (DOCX)

S3 Table Results of other types of feature selection. (DOCX)

S1 Doc Results of other types of classifier. (DOCX)

S1 Fig Binarized image of handwritten character and its Betti numbers. (DOCX)
