# Supplementary material for: Automated prediction of emphysema visual score using homology-based quantification of low-attenuation lung region
Source: PLoS One. 2017 May 25;12(5):e0178217. doi: 10.1371/journal.pone.0178217 (PMC5444793; doi:10.1371/journal.pone.0178217)
Supplement: S2 Table — (DOCX) [file pone.0178217.s002.docx]

**S2 Table.** Results of feature selection in C_LAA%_ and C_HEQ._

**(A)**

The predictive accuracy of C_LAA%_ when the length of feature vector was reduced to 10% of the original.

|  |  |  | lower limit of threshold (HU) | | | |  |
| --- | --- | --- | --- | --- | --- | --- | --- |
|  |  | −1000 | −950 | −900 | −850 | −800 | −750 |
|  | −950 | 42.6% |  |  |  |  |  |
|  | −900 | 54.8% | 51.3% |  |  |  |  |
| upper limit of threshold (HU) | −850 | 53.0% | 53.9% | 53.9% |  |  |  |
|  | −800 | 53.9% | 53.9% | 50.4% | 52.2% |  |  |
|  | −750 | 51.3% | 54.8% | 53.0% | 52.2% | 43.5% |  |
|  | −700 | 51.3% | 53.0% | 51.3% | 47.8% | 47.8% | 43.5% |
|  |  |  |  |  |  |  |  |

Note: The best accuracy was 54.8%; C_LAA%_, classifier using percentage of low-attenuation lung area as feature vector.

**(B)**

The predictive accuracy of C_LAA%_ when the length of feature vector was reduced to 30% of the original.

|  |  |  | lower limit of threshold (HU) | | | |  |
| --- | --- | --- | --- | --- | --- | --- | --- |
|  |  | −1000 | −950 | −900 | −850 | −800 | −750 |
|  | −950 | 48.7% |  |  |  |  |  |
|  | −900 | 56.5% | 55.7% |  |  |  |  |
| upper limit of threshold (HU) | −850 | 53.9% | 53.0% | 52.2% |  |  |  |
|  | −800 | 53.9% | 53.0% | 51.3% | 51.3% |  |  |
|  | −750 | 50.4% | 53.0% | 50.4% | 53.0% | 47.8% |  |
|  | −700 | 51.3% | 52.2% | 52.2% | 48.7% | 49.6% | 47.8% |
|  |  |  |  |  |  |  |  |

Note: The best accuracy was 56.5%; C_LAA%_, classifier using percentage of low-attenuation lung area as feature vector.

**(C)**

The predictive accuracy of C_LAA%_ when the length of feature vector was reduced to 50% of the original.

|  |  |  | lower limit of threshold (HU) | | | |  |
| --- | --- | --- | --- | --- | --- | --- | --- |
|  |  | −1000 | −950 | −900 | −850 | −800 | −750 |
|  | −950 | 50.4% |  |  |  |  |  |
|  | −900 | 54.8% | 55.7% |  |  |  |  |
| upper limit of threshold (HU) | −850 | 55.7% | 52.2% | 53.0% |  |  |  |
|  | −800 | 51.3% | 52.2% | 51.3% | 48.7% |  |  |
|  | −750 | 53.9% | 52.2% | 51.3% | 47.0% | 46.1% |  |
|  | −700 | 50.4% | 53.9% | 48.7% | 50.4% | 48.7% | 47.0% |
|  |  |  |  |  |  |  |  |

Note: The best accuracy was 55.7%; C_LAA%_, classifier using percentage of low-attenuation lung area as feature vector.

**(D)**

The predictive accuracy of C_HEQ_ when the length of feature vector was reduced to 10% of the original.

|  |  |  | lower limit of threshold (HU) | | | |  |
| --- | --- | --- | --- | --- | --- | --- | --- |
|  |  | −1000 | −950 | −900 | −850 | −800 | −750 |
|  | −950 | 46.1% |  |  |  |  |  |
|  | −900 | 56.5% | 62.6% |  |  |  |  |
| upper limit of threshold (HU) | −850 | 63.5% | 60.9% | 57.4% |  |  |  |
|  | −800 | 64.3% | 62.6% | 54.8% | 50.4% |  |  |
|  | −750 | 62.6% | 63.5% | 57.4% | 53.9% | 50.4% |  |
|  | −700 | 61.7% | 64.3% | 59.1% | 53.0% | 55.7% | 53.0% |
|  |  |  |  |  |  |  |  |

Note: The best accuracy was 64.3%; C_HEQ_, classifier using homology-based emphysema quantification as feature vector.

**(E)**

The predictive accuracy of C_HEQ_ when the length of feature vector was reduced to 30% of the original.

|  |  |  | lower limit of threshold (HU) | | | |  |
| --- | --- | --- | --- | --- | --- | --- | --- |
|  |  | −1000 | −950 | −900 | −850 | −800 | −750 |
|  | −950 | 49.6% |  |  |  |  |  |
|  | −900 | 56.5% | 57.4% |  |  |  |  |
| upper limit of threshold (HU) | −850 | 61.7% | 63.5% | 56.5% |  |  |  |
|  | −800 | 65.2% | 63.5% | 57.4% | 52.2% |  |  |
|  | −750 | 61.7% | 62.6% | 57.4% | 55.7% | 54.8% |  |
|  | −700 | 63.5% | 65.2% | 57.4% | 53.9% | 53.9% | 56.5% |
|  |  |  |  |  |  |  |  |

Note: The best accuracy was 65.2%; C_HEQ_, classifier using homology-based emphysema quantification as feature vector.

**(F)**

The predictive accuracy of C_HEQ_ when the length of feature vector was reduced to 50% of the original.

|  |  |  | lower limit of threshold (HU) | | | |  |
| --- | --- | --- | --- | --- | --- | --- | --- |
|  |  | −1000 | −950 | −900 | −850 | −800 | −750 |
|  | −950 | 49.6% |  |  |  |  |  |
|  | −900 | 59.1% | 60.0% |  |  |  |  |
| upper limit of threshold (HU) | −850 | 61.7% | 61.7% | 53.9% |  |  |  |
|  | −800 | 65.2% | 65.2% | 59.1% | 54.8% |  |  |
|  | −750 | 63.5% | 64.3% | 60.0% | 56.5% | 51.3% |  |
|  | −700 | 61.7% | 64.3% | 57.4% | 55.7% | 53.9% | 53.0% |
|  |  |  |  |  |  |  |  |

Note: The best accuracy was 65.2%; C_HEQ_, classifier using homology-based emphysema quantification as feature vector.
