# Supplementary material for: Automated prediction of emphysema visual score using homology-based quantification of low-attenuation lung region
Source: PLoS One. 2017 May 25;12(5):e0178217. doi: 10.1371/journal.pone.0178217 (PMC5444793; doi:10.1371/journal.pone.0178217)
Supplement: S3 Table — (DOCX) [file pone.0178217.s003.docx]

**S3 Table. Results of other types of feature selection.**

The following results of other types of feature selection correspond to those of C_HEQ_ (accuracy = 66.1%).

Detailed Process of Machine learning

The values of *nb*_0_ and *nb*_1_ at the threshold levels ranging from −1000 HU to −700 HU were used as feature vector. Before feature selection, the length of feature vector was 120. Then, mRMR, MRMD, or LASSO was performed as feature selection. Random Forest algorithm was used as machine learning algorithm. The following values were used as hyperparameters of Random Forest: number of trees in the forest, 10, 100, or 1000; and number of features to consider when searching best split, (length of feature vector) × 0.1, 0.3, 0.5, 0.7, or 0.9. Optimal hyperparameters were selected. Results of prediction were obtained using leave-one-patient-out cross validation. Feature selection was performed using the data of training partition.

**(A)**

mRMR, Minimum Redundancy and Maximum Relevance

| Length of feature vector (percentage to original length) | Accuracy |
| --- | --- |
| 12 (10%) | 60.0% |
| 36 (30%) | 58.3% |
| 60 (50%) | 60.9% |

**(B)**

MRMD, Maximum-Relevance-Maximum-Distance

| Length of feature vector (percentage to original length) | Accuracy |
| --- | --- |
| 12 (10%) | 58.3% |
| 36 (30%) | 60.0% |
| 60 (50%) | 59.1% |

**(C)**

LASSO

When coefficient of LASSO was larger than the predefined threshold, the corresponding feature was selected for machine learning algorithm.

| Threshold | Accuracy |
| --- | --- |
| 0.1 × (mean of coefficients of LASSO) | 61.7% |
| 1.0 × (mean of coefficients of LASSO) | 67.0% |
| 3.0 × (mean of coefficients of LASSO) | 62.6% |
