# Supplementary material for: Automated prediction of emphysema visual score using homology-based quantification of low-attenuation lung region
Source: PLoS One. 2017 May 25;12(5):e0178217. doi: 10.1371/journal.pone.0178217 (PMC5444793; doi:10.1371/journal.pone.0178217)
Supplement: S1 Doc — (DOCX) [file pone.0178217.s004.docx]

**S1 Doc. Results of other types of classifier.**

The following results correspond to those of C_HEQ_ (accuracy = 66.1%).

The values of *nb*_0_ and *nb*_1_ at the threshold levels ranging from −1000 HU to −700 HU were used as feature vector. The length of feature vector was 120. Results of prediction were obtained using leave-one-patient-out cross validation.

**(A)**

SVM, support vector machine

RBF was used for kernel trick. The following values were used as hyperparameters of SVM: C, 1/256, 1/16, 1, 16, or 256; gamma, 1/256, 1/16, 1, 16, or 256. Optimal hyperparameters were selected.

Accuracy = 54.8%

**(B)**

LibD3C [1]

The implementation of LibD3C was obtained from <http://lab.malab.cn/soft/LibD3C/>. To obtain the accuracy of LibD3C under leave-one-patient-out cross validation, we used default parameter setting of this implementation, because the implementation does not provide the way to parameter tuning when leave-one-patient-out cross validation is used.

Accuracy = 54.8%

**References**

1. Lin C, Chen W, Qiu C, Wu Y, Krishnan S, Zou Q. LibD3C: ensemble classifiers with a clustering and dynamic selection strategy. Neurocomputing. 2014. January 10; 123:424–35.
