# Supplementary material for: Automated prediction of emphysema visual score using homology-based quantification of low-attenuation lung region
Source: PLoS One. 2017 May 25;12(5):e0178217. doi: 10.1371/journal.pone.0178217 (PMC5444793; doi:10.1371/journal.pone.0178217)

**S1 Fig**

Binarized image of handwritten character and its Betti numbers.

Note: Betti numbers of binarized image of handwritten character were calculated. In the images, *b*_0_ corresponds to the number of black regions; *b*_1_ corresponds to the number of white regions.

1. A

*b*_0_ = 2, *b*_1_ = 1


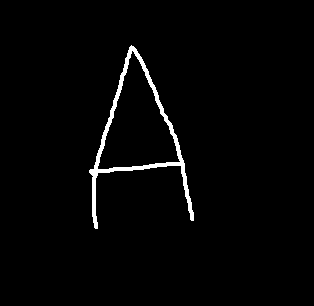


1. a

*b*_0_ = 2, *b*_1_ = 1


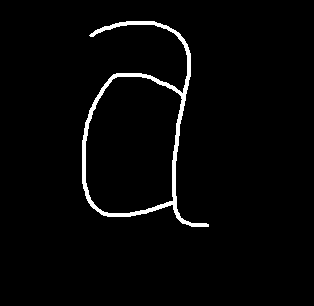


1. B

*b*_0_ = 3, *b*_1_ = 1


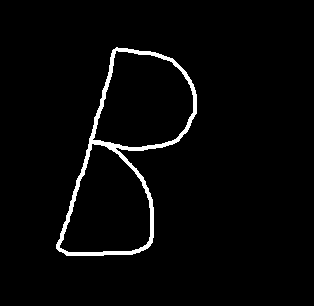


1. b

*b*_0_ = 2, *b*_1_ = 1


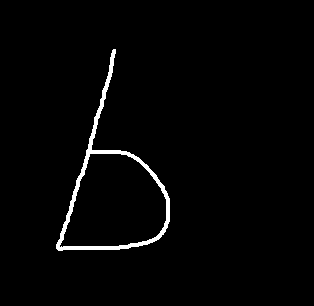


1. I

*b*_0_ = 1, *b*_1_ = 1


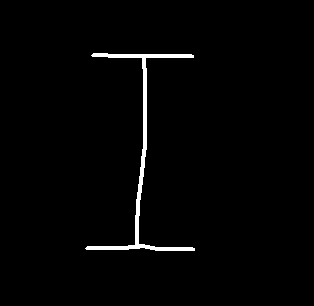


1. i

*b*_0_ = 1, *b*_1_ = 2


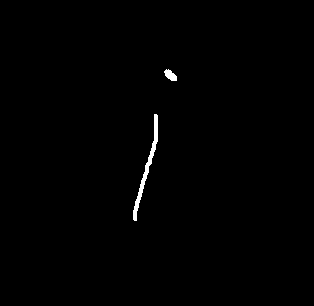

Supplement: S1 Fig — (DOCX) [file pone.0178217.s005.docx]
